# Failure of catecholamines to shift T-cell cytokine responses toward a Th2 profile in patients with rheumatoid arthritis

**DOI:** 10.1186/ar2028

**Published:** 2006-08-06

**Authors:** Matthias Wahle, Gesine Hanefeld, Stephan Brunn, Rainer H Straub, Ulf Wagner, Andreas Krause, Holm Häntzschel, Christoph GO Baerwald

**Affiliations:** 1 Department of Internal Medicine IV, University Hospital Leipzig, Liebigstrasse 22, 04103 Leipzig, Germany; 2 Laboratory of Experimental Rheumatology and Neuroendocrino-Immunology, Department of Internal Medicine I, University Hospital Regensburg, Franz-Josef-Strauss-Allee 11, 93042 Regensburg, Germany; 3 Immanuel Hospital, Rheumatology Clinic, Königstrasse 63, 14109 Berlin, Germany

## Abstract

To further understand the role of neuro-immunological interactions in the pathogenesis of rheumatoid arthritis (RA), we studied the influence of sympathetic neurotransmitters on cytokine production of T cells in patients with RA. T cells were isolated from peripheral blood of RA patients or healthy donors (HDs), and stimulated via CD3 and CD28. Co-incubation was carried out with epinephrine or norepinephrine in concentrations ranging from 10^-5 ^M to 10^-11 ^M. Interferon (IFN)-γ, tumour necrosis factor (TNF)-α, interleukin (IL)-4, and IL-10 were determined in the culture supernatant with enzyme-linked immunosorbent assay. In addition, IFN-γ and IL-10 were evaluated with intracellular cytokine staining. Furthermore, basal and agonist-induced cAMP levels and catecholamine-induced apoptosis of T cells were measured. Catecholamines inhibited the synthesis of IFN-γ, TNF-α, and IL-10 at a concentration of 10^-5 ^M. In addition, IFN-γ release was suppressed by 10^-7 ^M epinephrine. Lower catecholamine concentrations exerted no significant effect. A reduced IL-4 production upon co-incubation with 10^-5 ^M epinephrine was observed in RA patients only. The inhibitory effect of catecholamines on IFN-γ production was lower in RA patients as compared with HDs. In RA patients, a catecholamine-induced shift toward a Th2 (type 2) polarised cytokine profile was abrogated. Evaluation of intracellular cytokines revealed that CD8-positive T cells were accountable for the impaired catecholaminergic control of IFN-γ production. The highly significant negative correlation between age and catecholamine effects in HDs was not found in RA patients. Basal and stimulated cAMP levels in T-cell subsets and catecholamine-induced apoptosis did not differ between RA patients and HDs. RA patients demonstrate an impaired inhibitory effect of catecholamines on IFN-γ production together with a failure to induce a shift of T-cell cytokine responses toward a Th2-like profile. Such an unfavorable situation is a perpetuating factor for inflammation.

## Introduction

Rheumatoid arthritis (RA) is a chronic inflammatory disease characterised by intense immune activation within the synovial compartment of joints and a variety of systemic manifestations. The inflammatory process leads to cartilage and bone destruction [1]. Although the pathophysiology of RA is not completely understood, the abundance of T cells within the mononuclear infiltrates of the hyperplastic synovial membrane in RA together with the local production of T cell-derived cytokines suggest that T cells are important in the autoimmune response in RA [2]. According to the cytokine profiles after activation, CD4-positive T cells are subdivided into different subclasses termed T helper lymphocyte type 1 (Th1), Th2, and others [3]. Th1 and Th2 subsets can be viewed as the polarised accentuation of an immune reaction determining the local cytokine milieu [3]. Importantly, Th1 cells inhibit the generation of Th2 cells and *vice versa*. RA is interpreted as a disease dominated by a Th1 response and selective accumulation of Th1 cells within the synovial compartment [4]. Although local Th1 cell activation is regarded as the most important mechanism in enhancing inflammation during the course of RA [5], CD8-positive T cells are supposed to play an important role in the distinct pathology of RA as well [6].

Although the etiology of RA remains elusive, the hallmark of the clinical course is a symmetric arthritis. Since the clinical observation that paralysed joints in patients who had an upper motor neuron hemiplegia or poliomyelitis were spared from the inflammatory process [7], an important role for the nervous system in the pathogenesis of RA has been hypothesised. It is proposed that in rheumatic diseases a disturbed interaction of the sympathetic nervous system (SNS) and the immune system contributes to the pathogenic process [8]. In particular, a dysbalance between the pro-inflammatory influence of substance P released by afferent sensory nerve fibers and the anti-inflammatory effect of norepinephrine (NE) released by efferent sympathetic nerve fibers is proposed in RA [9]. In addition, chronic inflammatory diseases such as RA, juvenile chronic arthritis, and multiple sclerosis (MS) are frequently accompanied by clinical symptoms of altered sympathetic activity [10,11].

The requirements for sympathetic neural interactions with lymphoid and accessory cells of the immune system are fulfilled because (a) lymphoid tissue is densely innervated by the SNS, (b) neurotransmitters are released by neural varicosities, (c) cells of the immune system express adrenergic receptors, mainly of the β2-adrenergic type (β2R), and (d) a robust response of immune cells can be detected after catecholamine release [12]. The physiological role of the SNS in the generation of an immune response is not yet fully understood. Fine-tuning of the magnitude and/or the duration of an immune response is the most favored hypothesis. A recent study demonstrates pro-inflammatory actions of the SNS during the induction phase of adjuvant arthritis and an anti-inflammatory role in the effector phase [13].

To further investigate the impact of catecholamines on cytokine production of human T lymphocyte populations of age-matched healthy donors (HDs) and patients with RA, peripheral circulating T cells were activated and the production of the cytokines interleukin (IL)-4, IL-10, interferon (IFN)-γ, and tumour necrosis factor (TNF)-α was studied upon co-incubation with epinephrine (EPI) or NE. In addition, signal transduction of β2R was determined using cAMP as the readout parameter.

## Materials and methods

### Study population and determination of disease activity

Sixteen consecutive patients with RA according to the revised American College of Rheumatology criteria [14] and a group of 16 age-matched healthy blood donors were included in the study. To exclude a potential influence of therapy with disease-modifying anti-rheumatic drugs (DMARDs) on β2R characteristics, only patients without current DMARD therapy were included in the study. In addition, therapy with TNF-α blocking agents or other biologicals was not allowed. Furthermore, we excluded patients in whom other factors were supposed to influence β2R (that is, infectious and atopic diseases, hyperthyroidism or hypothyroidism, untreated hypertension, therapy with sympathomimetics or sympatholytics, and cancer). Patients were examined by taking history, physical examination, and laboratory findings (erythrocyte sedimentation rate, C-reactive protein [CRP], rheumatoid factor, anti-nuclear antibodies, hemoglobin, leukocytes, lymphocytes, platelets, and creatinine). Inflammatory disease activity in RA was determined by the DAS28-3 (Disease Activity Score using 28 joints and three variables) [15]. The clinical characteristics of patients and control subjects are summarised in Table 1. Tests for antibodies to cyclic citrullinated peptides (anti-CCP antibodies) of nine patients with RA were available. Seven patients with RA were positive for anti-CCP antibodies, whereas the remaining two demonstrated a negative result. Treatment with non-steroidal anti-rheumatic drugs or glucocorticoids up to 7.5 mg prednisolone equivalent per day was allowed in the patient group (four of 16 patients with RA, range 2 to 7.5 mg prednisolone equivalent per day). Previous investigations revealed that β2R characteristics are not influenced by corticosteroids at this dosage [16,17].

The study protocol was approved by our local ethics committee, and informed consent was obtained from all subjects included in the study.

### Separation of T lymphocytes and cell culture

Peripheral blood mononuclear cells (PBMCs) of patients with RA and HDs were separated from peripheral venous blood by Ficoll-Hypaque (Biochrom AG, Berlin, Germany) density gradient centrifugation. CD3-, CD4-, or CD8-positive T cells were isolated using the MACS (magnetic activated cell sorting) technique (CD3 microbeads, CD4 and CD8 T-cell isolation kit; Miltenyi Biotec GmbH, Bergisch Gladbach, Germany) as described earlier [18,19]. The purity of the isolated T cells was evaluated with flow cytometry and exceeded 95% in each experiment. A serum-free culture medium (RPMI 1640 supplemented with 100 IU/ml penicillin, 100 μg/ml streptomycin, 2 mM l-glutamine, and 2% TCH defined serum supplement; MP Biochemicals, Heidelberg, Germany) was used throughout the study. Cells (1 × 10^6^/cells in 2 ml culture medium) were cultured at 37°C and 5% CO_2 _in a humidified atmosphere. Mitogenic stimulation of Tcells was performed with plate-bound anti-CD3-monoclonal antibody (mAb) (clone UCHT-1, 10 μg/ml), anti-CD28-mAb (clone 37.407.111, 2 μg/ml), and recombinant human IL-2 (0.5 ng/ml; all from R&D Systems GmbH, Wiesbaden-Nordenstadt, Germany). For the detection of intracellular cytokine content, T cells were stimulated for 12 hours in the presence of 2 mM Brefeldin A (Sigma-Aldrich, St. Louis, MO, USA). Co-incubation was carried out with EPI- and NE-hydrochloride (10^-5 ^to 10^-11 ^M; Aventis-Pharma GmbH, Frankfurt, Germany). The supposed concentrations of NE in the vicinity of sympathetic nerve fibers in lymphoid organs and the average plasma concentration of EPI and NE were reported to be in the range of 10^-5 ^and 10^-9 ^M, respectively [20,21]. Blocking of specific catecholaminergic effects was carried out by parallel addition of the β-adrenergic receptor blocker propranolol (10^-5 ^M; Schwarz Pharma AG, Monheim, Germany).

### Determination of cytokines

In a preliminary set of experiments, the kinetic of IFN-γ and IL-10 production was determined in six HDs and seven patients with RA at 24, 48, or 72 hours. Both cytokines were then determined in 16 HDs and patients with RA at 48 hours. The synthesis of TNF-α and IL-4 was measured in six HDs and seven patients with RA. Cell culture supernatants of stimulated T cells were collected and immediately analysed or stored frozen at -80°C until analysis. IFN-γ, TNF-α, IL-4, and IL-10 enzyme-linked immunosorbent assay (ELISA) kits (OptEIA Immunoassay kit) were purchased from BD Biosciences (Becton Dickinson GmbH, Heidelberg, Germany). The samples and standards were diluted in assay diluent (phosphate-buffered saline [PBS] supplemented with 10% fetal calf serum [FCS]) and analysed in duplicate. All procedures were followed according to the recommendations of the manufacturer. The range of cytokine detection was as follows: IFN-γ (range 4.7 to 300 pg/ml), TNF-α (range 4.7 to 300 pg/ml), IL-4 (range 4.7 to 300 pg/ml), and IL-10 (range 7.8 to 500 pg/ml). The intra- and interassay coefficients of variation were less than 10%.

### Evaluation of cell surface antigens

Aliquots of isolated T cells were washed in PBS and stained using anti-CD3-fluorescein isothiocyanate (FITC) (clone UCHT-1; Dako Deutschland GmbH, Hamburg, Germany), anti-CD8-phycoerythrin (PE) (clone B9.11; Beckman Coulter GmbH, Krefeld, Germany), and anti-CD4-PC5 (clone 13B82; Beckman Coulter) mAbs for 30 minutes at 4°C. T-cell purity and the CD4/CD8 ratio were determined after a final wash step in PBS with a FACSCalibur (BD Biosciences) and CellQuest Pro software (BD Biosciences).

### Determination of intracellular cytokines

Intracellular IFN-γ and IL-10 were determined in stimulated CD3-positive lymphocytes of five HDs and five patients with RA as described [22]. Briefly, CD4 and CD8 were stained with the appropriate mAb (CD4: PC5-coupled, clone 13B82; Beckman Coulter; CD8: allophycocyanin-conjugated, clone RPA-T8; BD Biosciences). Cells were then fixed with 2% paraformaldehyde in PBS, permeabilised with Saponine-buffer (PBS, 2% FCS, 0.1% Saponine; Sigma-Aldrich), and incubated with FITC-coupled anti-IFN-γ (clone 4S.B3; BD Biosciences) and PE-coupled anti-IL-10 (clone JES3-19F1; BD Biosciences) mAbs for 30 minutes at 4°C. At least 10,000 events were counted for each experiment. IFN-γ- or IL-10-producing cells were analysed in the CD4-positive and CD8-positive T-cell subpopulations after applying a constant gate that was set on the respective marker. The percentage of positive cells was determined with CellQuest Pro software, using two-dimensional dot plots.

### Determination of apoptosis

The proportion of apoptotic cells was evaluated in isolated Tcells of five patients with RA and five HDs. T cells were stimulated for 48 hours, and co-incubation was carried out with EPI or NE (10^-5 ^and 10^-9 ^M). Cells were washed in Annexin binding buffer (150 mM NaCl, 10 mM HEPES, and 2 mM CaCl_2_) and stained with Annexin-V-FITC (Bender MedSystems GmbH, Vienna, Austria), propidium iodide (PI), CD8-PE (clone B9.11; Beckman Coulter), and CD4-APC (clone RPA-T4; BD Biosciences) for 30 minutes at 4°C. The proportion of early (Annexin-V-positive/PI-negative) and late apoptotic/necrotic (Annexin-V-positive/PI-positive) cells was determined in the CD4-positive and CD8-positive subpopulations, using two-dimensional dot blots and appropriate gates.

### Evaluation of basal and stimulated intracellular cAMP

Basal and stimulated levels of cAMP were determined in CD4-positive and CD8-positive T cells (HDs, *n *= 8; patients with RA, *n *= 5). Aliquots of 2 × 10^6 ^cells in incubation buffer (PBS, 0.5% bovine serum albumin, 250 μM ascorbic acid, and 100 μM theophylline) were incubated for 10 minutes at 37°C in a water bath. The β2R agonist terbutaline (10^-5 ^M; Sigma-Aldrich) or incubation buffer was added, and the cells were incubated for an additional 15 minutes at 37°C. Incubation buffer was then added in excess to terminate the reaction. Cells were then lysed by the addition of 150 μl 0.1 N hydrochloric acid. The concentrations of baseline and stimulated levels of cAMP in CD4- and CD8-positive T cells were determined using a cAMP ELISA (low pH; R&D Systems GmbH) according to the guidelines of the manufacturer.

### Statistical analysis

Values in the table and figures are given in means and standard errors of the mean (if not otherwise indicated). The relative change of cytokine production upon co-incubation with catecholamines was determined after defining the concentration of the respective cytokine in the control cultures as 100% for each patient and healthy control. A comparison of the catecholamine effect on cytokine production was calculated by the repeated measures analysis of variance (ANOVA) followed by the Bonferroni test. When the normality test failed, the Kruskal-Wallis test and the Dunnett's method for calculation of multiple comparisons were used. The relative catecholamine response values in comparison between patients with RA and controls were first analysed by the one-way ANOVA to determine whether an overall statistically significant change existed before using the two-tailed unpaired Student's *t *test. A correlation analysis between disease characteristics and cytokine concentrations was carried out by means of Pearson product moment or the Spearman rank order correlation. Statistically significant differences were considered when *p *< 0.05.

## Results

### Influence of catecholamines on cytokine production by T cells

The preliminary experiments determining the kinetics of IFN-γ and IL-10 production in patients with RA demonstrated an increase of IFN-γ production in the first 48 hours in HDs (24 hours: 658 ± 221 pg/ml; 48 hours: 6,195 ± 1,920 pg/ml) and a slight decrease thereafter (72 hours: 5,952 ± 3,030 pg/ml). IL-10 production increased over the entire culture period (24 hours: 112 ± 38 pg/ml; 48 hours: 412 ± 179 pg/ml; 72 hours: 496 ± 155 pg/ml). Patients with RA exhibited increasing IFN-γ (24 hours: 73 ± 10 pg/ml; 48 hours: 670 ± 242 pg/ml; 72 hours: 1,867 ± 596 pg/ml) as well as IL-10 synthesis (24 hours: 21 ± 13 pg/ml; 48 hours: 166 ± 68 pg/ml; 72 hours: 240 ± 72 pg/ml). IFN-γ and IL-10 synthesis was lower in patients with RA compared with HDs at each time point studied (*p *< 0.05, two-way ANOVA). The relative influence of 10^-5 ^M EPI on IFN-γ production differed significantly between patients with RA and HDs at 24 hours (patients with RA 74% ± 10%, HDs 27% ± 8% of control cultures, *p *< 0.01). NE or lower concentrations of EPI demonstrated no overt differences regarding the influence on IFN-γ production. Catecholamines showed no difference on IL-10 synthesis at the different time points or between HDs and patients with RA (data not shown).

**Figure 1:**
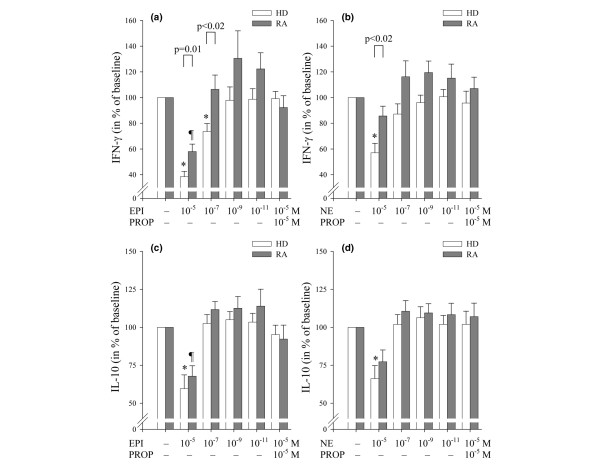
Modulation of interferon (IFN)-γ and interleukin (IL)-10 synthesis of activated T cells by catecholamines in healthy donors (HDs) (*n *= 16, white bars) and patients with rheumatoid arthritis (RA) (*n *= 16, gray bars). The concentrations of IFN-γ upon co-incubation with **(a) **epinephrine (EPI) and **(b) **norepinephrine (NE) and of IL-10 upon co-incubation with **(c) **EPI and **(d) **NE were determined in the culture supernatant by means of enzyme-linked immunosorbent assay after T-cell stimulation for 48 hours. The baseline values of each RA patient and HD were defined as 100%, and the cytokine concentrations upon co-incubation with catecholamines were expressed as percentage of baseline values. *p < 0.001 and ^¶^*p *< 0.05 denote significant differences in cytokine production of patients with RA or HDs compared with the control values. *P *values indicate significant differences between the catecholamine effects of patients with RA and HDs, respectively. PROP, propranolol.

Because the cytokine synthesis of patients with RA was very low at 24 hours, and even further reduced by catecholamines, final cytokine analysis was carried out at 48 hours. High concentrations of EPI or NE (10^-5 ^M) significantly inhibited IFN-γ production in HDs (baseline values of IFN-γ: HDs 3,461 ± 960 pg/ml; patients with RA 2,117 ± 568 pg/ml; *p *= 0.396) (Figure 1a). IL-10 synthesis was suppressed by 10^-5 ^M EPI, but not by NE (baseline values of IL-10: HDs 322 ± 95 pg/ml; patients with RA 148 ± 34 pg/ml; *p *= 0.165) (Figure 1c,d). Lower concentrations of catecholamines exerted no significant effect. In patients with RA, 10^-5 ^M EPI significantly inhibited IFN-γ and IL-10 expression at 48 hours whereas NE did not suppress cytokine production significantly (Figure 1). In addition, the reduction of IFN-γ synthesis was significantly lower in patients with RA upon co-incubation with 10^-5 ^M EPI or NE and 10^-7 ^M EPI (Figure 1a, b). In contrast to IFN-γ production, that of IL-10 was similarly affected by catecholamines in HDs and patients with RA (Figure 1c,d).

**Figure 2:**
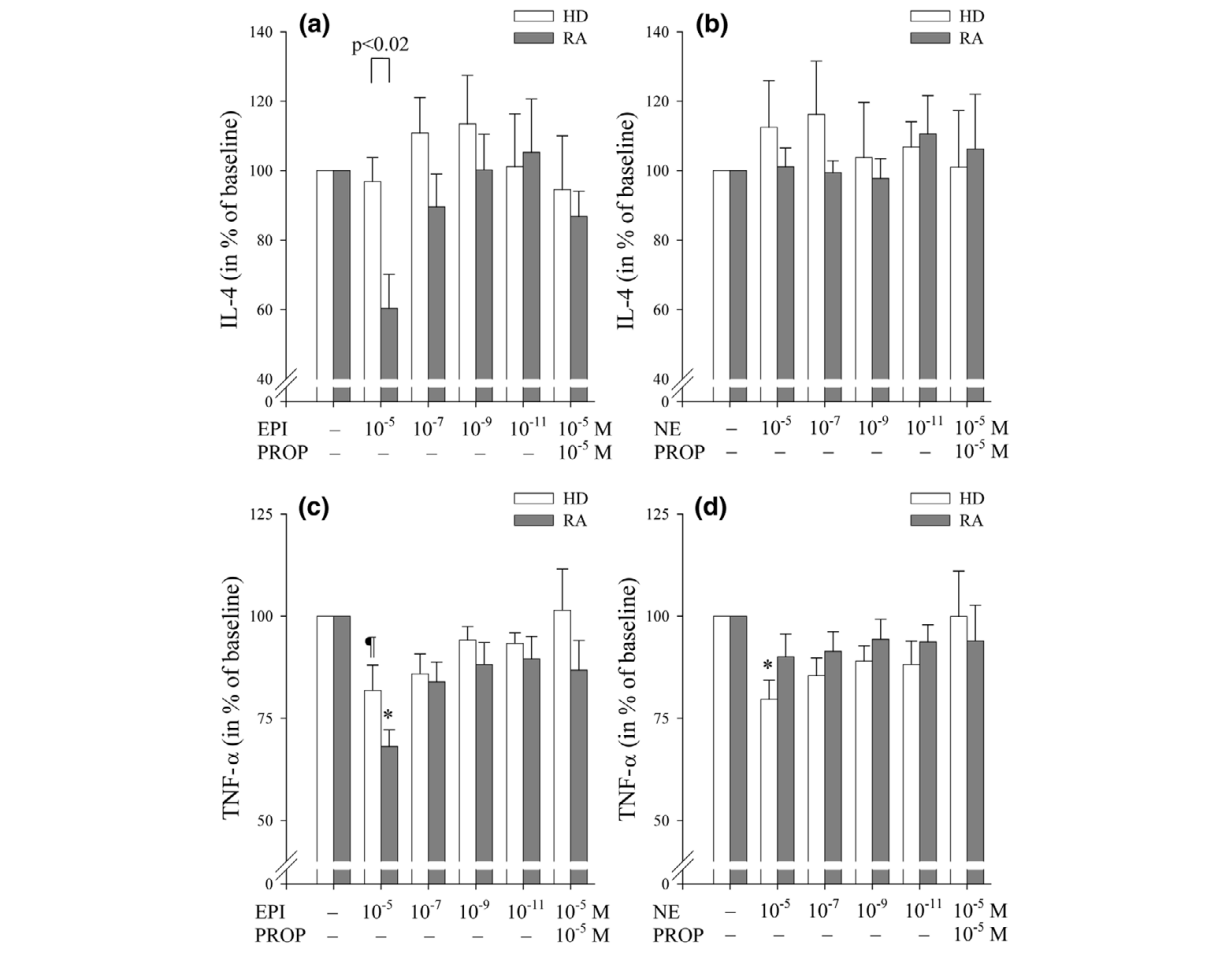
Modulation of interleukin (IL)-4 and tumour necrosis factor (TNF)-α synthesis of activated T cells by catecholamines in healthy donors (HDs) (IL-4: *n *= 6; TNF-α: *n *= 5, white bars) and patients with rheumatoid arthritis (RA) (IL-4: *n *= 5; TNF-α: *n *= 7, gray bars). The concentrations of IL-4 upon co-incubation with **(a) **epinephrine (EPI) and **(b) **norepinephrine (NE) and of TNF-α upon co-incubation with **(c) **EPI and **(d) **NE were determined in the culture supernatant by means of enzyme-linked immunosorbent assay after T-cell stimulation for 48 hours. The baseline values of each HD and patient with RA were defined as 100%, and the cytokine concentrations upon co-incubation with catecholamines are given as percentage of baseline values. *p < 0.001 and ^¶^*p *< 0.05 as compared with values obtained upon stimulation in the presence of medium alone. *P *values indicate significant differences between the catecholamine effects of patients with RA and HDs, respectively. PROP, propranolol.

IL-4 secretion of activated T cells from HDs was not influenced by catecholamines (baseline values of IL-4: HDs 365 ± 62 pg/ml; patients with RA 300 ± 95 pg/ml; *p *= 0.579) (Figure 2a,b). In patients with RA, the IL-4 concentration in the culture supernatant of activated T cells was suppressed by 10^-5 ^M EPI, just failing to reach the significance level compared with control cultures (*p *= 0.055). However, the relative production of IL-4 upon the influence of 10 μM EPI was significantly lower in patients with RA compared with HDs (*p *< 0.02, Figure 2a).

TNF-α synthesis was suppressed dose-dependently by catecholamines. A significant inhibition was observed upon co-incubation with 10^-5 ^M EPI or NE in HDs and 10^-5 ^M EPI in patients with RA at 48 hours (baseline levels of TNF-α: HDs 3,524 ± 554 pg/ml; patients with RA 2,087 ± 432 pg/ml; *p *= 0.065) (Figure 2c,d). No difference was observed between the relative inhibition of TNF-α by catecholamines in patients with RA and HDs (Figure 2c, d). Incubation of activated T cells with 10^-5 ^M propranolol in parallel to 10^-5 ^M EPI or NE antagonised the catecholamine effects on cytokine production (Figures 1 and 2).

**Figure 3:**
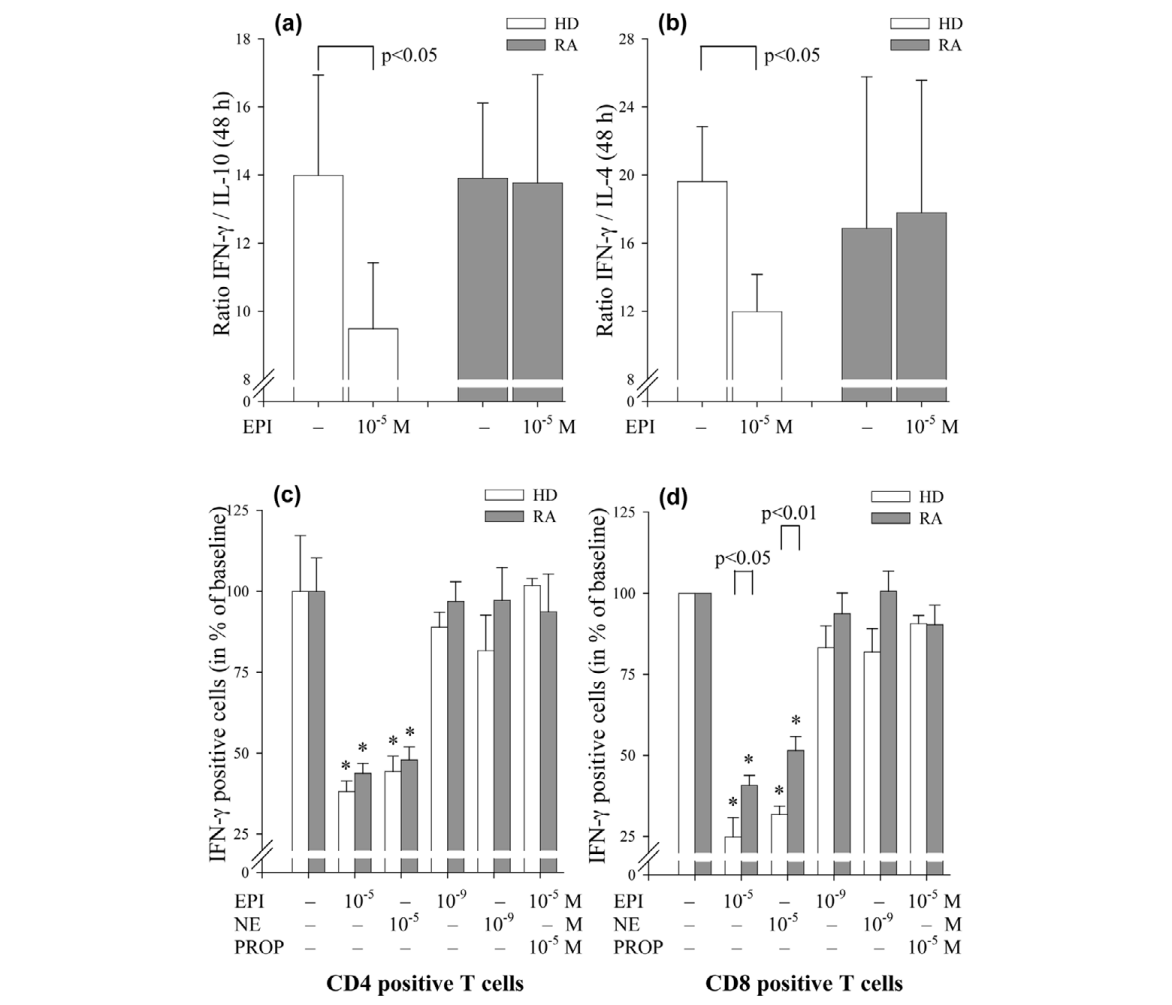
Influence of catecholamines on cytokine ratios and intracellular interferon (IFN)-γ expression in healthy donors (HDs) (white bars) and patients with rheumatoid arthritis (RA) (gray bars). The ratios between the raw cytokine values of **(a) **IFN-γ and interleukin (IL)-10 (*n *= 16) or **(b) **IFN-γ and IL-4 (HDs: *n *= 6; patients with RA: *n *= 5) were evaluated after stimulation of T cells for 48 hours with medium and 10^-5 ^M epinephrine (EPI), respectively. Intracellular IFN-γ was determined in **(c) **CD4-positive and **(d) **CD8-positive T cells activated for 12 hours. The baseline values of IFN-γ-positive cells in the CD4 and CD8 T-cell populations of each RA patient and HD were defined as 100%, and the number of IFN-γ-positive cells upon co-incubation with catecholamines related to the baseline values of each HD and patient with RA. *p < 0.05 as compared with the values obtained upon control conditions. NE, norepinephrine; PROP, propranolol.

Examination of the cytokine ratio of activated T cells from HDs revealed a significant decrease in the IFN-γ/IL-10 ratio upon co-incubation with 10^-5 ^M EPI (Figure 3a). Likewise, the IFN-γ/IL-4 ratio decreased upon co-incubation with 10^-5 ^M EPI (Figure 3b). However, in patients with RA, EPI failed to induce any shift in the IFN-γ/IL-10 or IFN-γ/IL-4 ratios (Figure 3a,b).

### Influence of catecholamines on intracellular cytokine expression

The number of IFN-γ-producing cells was higher in the CD8-positive compared with the CD4-positive population (HDs: 6.6% ± 1.1% CD4/IFN-γ-positive cells and 14.6% ± 2.2% CD8/IFN-γ-positive cells, *p *< 0.02; patients with RA: 2.7 ± 0.3 CD4/IFN-γ-positive cells and 6.2 ± 0.4 CD8/IFN-γ-positive cells, *p *< 0.001). In addition, increased numbers of CD4/IFN-γ- and CD8/IFN-γ-positive T cells were detected in the control subjects versus patients with RA (*p *< 0.05, Figure 3c, d). High-dose EPI or NE (10^-5 ^M) significantly inhibited the expression of intracellular IFN-γ in CD4- and CD8-positive T cells of patients with RA and controls (Figure 3c, d). The inhibition of IFN-γ expression in CD4-positive T cells did not differ between patients with RA and controls (Figure 3c). However, the relative reduction in the number of CD8-positive/IFN-γ cells was significantly more pronounced in HDs compared with patients with RA (Figure 3d). Low concentrations of catecholamines exerted no significant effect. The functional effects of 10^-5 ^M catecholamines could be abrogated by propranolol (Figure 3c, d).

### Influence of age and disease activity on cytokine production by T cells and catecholamine effects

**Figure 4:**
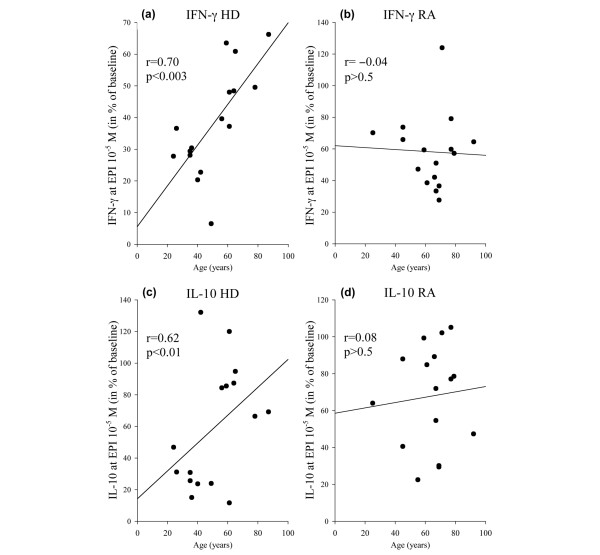
Correlation between age and epinephrine (EPI) effects in patients with rheumatoid arthritis (RA) and healthy subjects. The age of the subjects was plotted against concentrations of the relative interferon (IFN)-γ in **(a) **healthy donors (HDs) and **(b) **patients with RA and of interleukin (IL)-10 in **(c) **HDs and **(d) **patients with RA after co-incubation of activated T cells with 10^-5 ^M EPI for 48 hours. The baseline values of each HD and patient with RA were defined as 100%. (Concentrations are presented in Figure 1 legend.) The cytokine concentrations upon co-incubation with 10^-5 ^M EPI are given as percentage of baseline values. p, the *p *value; r, the respective correlation coefficient.

In HDs, a significant positive correlation existed between age and the relative concentrations of IFN-γ produced by activated T cells after co-incubation with 10^-5 ^M EPI (Figure 4a), indicating an age-related decline of the suppressive effect of EPI on IFN-γ production (increased values mean less suppression). A similar relationship was observed between the effect of EPI upon IL-10 synthesis of activated T cells and age in HDs (Figure 4c). The correlation between age and NE effects and the correlation between age and effects at lower concentrations of EPI (10^-7 ^to 10^-11 ^M) remained non-significant (data not shown).

Interestingly, in patients with RA, no such correlation was evident between age and effects of EPI at the high concentrations (Figure 4b, d). Additionally, no correlation was observed between baseline cytokine values or the functional effect of catecholamines and disease activity, disease duration, the number of involved joints, CRP, and other laboratory values. Furthermore, no relationship was observed between baseline cytokine values or catecholamine effects and the CD4/CD8 ratio or the amount of corticosteroids administered.

### Generation of cAMP after stimulation of β2R of T cells

Stimulation of β2R with terbutaline induced cAMP accumulation in isolated CD4- and CD8-positive T cells of patients with RA and HDs (data not shown). However, no significant differences were found regarding agonist-induced cAMP generation of T-cell subpopulations in patients with RA compared with HDs (data not shown).

### Influence of catecholamines on T-cell apoptosis

Given that catecholamines have been reported to induce apoptosis in T lymphocytes [23], the possible influence of cell death on cytokine production of stimulated T cells upon co-incubation with catecholamines was tested. After stimulation of T cells for 48 hours with anti-CD3-mAb and anti-CD28-mAb together with IL-2, 2.3% ± 0.3% T cells went into apoptosis (Annexin-V-positive, PI-negative) in HDs compared with 2.6% ± 0.5% in patients with RA (*p *= 0.674). The proportion of late apoptotic/necrotic cells was 6.2% ± 0.7% in HDs and 10.3% ± 2.6% in patients with RA (*p *= 0.337). In the presence of 10^-5 ^M and 10^-9 ^M EPI or NE, no change in the number of apoptotic or necrotic cells was observed in HDs and patients with RA (data not shown).

## Discussion

In the present study, we determined the effects of catecholamines on cytokine production of T cells from patients with RA and HDs. In addition, cAMP generation and induction of apoptosis after β2R stimulation were evaluated. The functional effects of catecholamines on cytokine production demonstrate an impaired suppression of IFN-γ production in patients with RA together with a failure to induce a shift toward a more Th2-like cytokine profile that is observed in HDs. Moreover, the reduced catecholaminergic control on IFN-γ production in patients with RA compared with HDs mainly affects CD8-positive T cells. The functional discrepancy of cytokine synthesis in patients with RA versus HDs upon the influence of catecholamines is cytokine-specific. Whereas the production of IFN-γ is inhibited to a lesser extent in patients with RA, the catecholaminergic influence on IL-10 and TNF-α is unaffected. Otherwise, a catecholamine-induced suppression of IL-4 production by T cells is observed only in patients with RA.

Catecholamines regulate cytokine expression depending on the cytokine studied, the cell type, and the experimental conditions used. In general, the production of Th1 (type 1) cytokines and TNF-α is inhibited, whereas Th2 (type 2) cytokines are slightly suppressed, unchanged, or even induced by catecholamines (reviewed in [24]). However, under conditions promoting Th1 development (that is, stimulation in the presence of IL-12), it could be demonstrated that high doses of NE (10 μM) increase expression of the prototypic Th1 cytokine IFN-γ in CD4-positive T cells [25].

Little is known about the impact of catecholamines on cytokine production in patients with RA. A comparable increase of IFN-γ-producing lymphocytes in peripheral blood was induced by EPI infusion in patients with RA and HDs [26]. The recruitment of IFN-γ-producing lymphocytes into the periphery rather than a true increase in cytokine synthesis by EPI has been proposed [26]. In contrast, the number and EPI-induced increase of IL-10-positive monocytes were higher in patients with RA compared with HDs [26]. After induction of mental stress by public speaking, a significant increase of IFN-γ production upon stimulation of PBMCs with phytohaemagglutinin was detected in HDs but not in patients with RA [27]. Suppression of TNF-α synthesis by the β2R agonist terbutaline was higher in lipopolysaccharide-stimulated PBMCs of patients with RA *in vitro *[28]. In the collagen type II-induced arthritis model of mice, chemical sympathectomy before the induction of arthritis decreased disease severity and induced the expression of IL-4 and IL-10. In contrast, sympathectomy in the chronic phase aggravated the inflammatory process and also resulted in an increase in IFN-γ and TNF-α [13].

The results of our study point to a similar mechanism demonstrating a disturbed interaction of the SNS and the immune system. Although catecholamines shift the cytokine profile toward a Th2-dominated cytokine environment in HDs, the IFN-γ/IL-10 ratio or IFN-γ/IL-4 ratio does not change after co-incubation with EPI in patients with RA. Hence, the outcome after physiological or pharmacological β2R stimulation results in a pro-inflammatory rather than an anti-inflammatory cytokine environment in patients with RA [29]. A similar reduction in the catecholaminergic control of IFN-γ expression was recently reported in patients with MS [30]. In addition, the same study demonstrated a restored suppression of IFN-γ expression in patients with MS treated with IFN-β. A disruption of the β2R-mediated suppression of IFN-γ production has been shown after allergen provocation in patients with asthma whereas the reactivity via prostaglandin E2 (PGE2) remained unchanged [31].

Of interest, a highly significant correlation between age and suppression of IFN-γ and IL-10 was observed in HDs. This relationship was completely abrogated in patients with RA. An accelerated aging of the immune system has been described in RA [32]. As an example, immunosenescence in healthy subjects and premature aging of the immune system in RA are characterised by shrinkage of the T-cell receptor repertoire and loss of costimulatory molecules such as CD28 [33]. Most likely, the inflammatory process in RA disrupts the age-related control of catecholamines on cytokine production. In this respect, alterations of the autonomic nervous system, including increased circulating NE concentrations and reduced β2R expression, are similar in elderly people as compared with patients with RA [33]. Consequently, the differences in the catecholamine-controlled cytokine production can be regarded as part of a vicious circle aggravating the inflammatory process that characterises the distinct pathology of RA. It is yet unknown whether the altered influence of catecholamines on T-cell cytokine production is specific for RA or MS. However, a comparable reduction in β2R expression on PBMCs has been observed in RA and other chronic inflammatory diseases like systemic lupus erythematosus (SLE) or chronic inflammatory bowel disease [16,34]. Hence, it might be suggested that the inflammatory process itself modifies β2R function. Nevertheless, an altered influence of catecholamines on cytokine production has to be interpreted in the context of a disease-specific cytokine profile (for example, more polarised Th1- or Th2-like reactions in RA and SLE, respectively).

From a molecular point of view, the functional effects of catecholamines on lymphocytes are induced by the accumulation of intracellular cAMP in lymphocytes after binding to constitutively expressed β2R coupled to stimulatory guanine nucleotide binding proteins (Gs-proteins) [35,36]. This in turn leads to an adenylyl cyclase-mediated cAMP increase, activation of protein kinase A (PKA) [37], and finally to binding of the transcription factor CREB (cAMP-responsive element binding protein) to specific cAMP-responsive elements on the DNA [38]. In the present study, β2R-mediated cAMP generation in CD4-positive and CD8-positive T cells was similar in patients with RA and HDs. In contrast, in PBMCs, previous investigations revealed an increased cAMP production and a higher activity of the PKA-I and -II isoenzymes in patients with RA [28,39]. On the other hand, B cells of patients with RA demonstrated a reduced agonist-induced cAMP generation [16]. These differences in catecholaminergic cAMP generation suggest that complex and cell-specific changes of β2R function and signal transduction account for alterations of catecholamine effects on lymphocyte function in RA.

The similar influence of catecholamines on TNF-α and IL-10 production in patients with RA and HDs is in agreement with the unchanged β2R agonist-induced cAMP generation in CD4- and CD8-positive T cells of patients with RA. An explanation for the enhanced catecholaminergic influence on IL-4 synthesis in patients with RA may be found in an increased inhibitory effect of catecholamines on IL-2 production that decreases IL-4 synthesis indirectly [40,41]. However, T cells were stimulated in the presence of IL-2 in our study. On the other hand, human Th1 cells produce low levels of IL-4 [42], and increased numbers of Th1 and Th0 cells have been reported in the peripheral circulation of patients with RA [43,44]. In contrast to Th2 cells, Th1 and Th0 cells express β2R [45]. Hence, the effect of EPI on IL-4 production most likely reflects a different T helper cell composition in the peripheral blood of patients with RA.

In any case, the reduced functional effect of catecholamines on IFN-γ production is explained neither by differences in cAMP generation nor by the frequency of Th-cell subtypes in patients with RA. Moreover, different functional effects of PGE2 and isoproterenol on T-cell proliferation, regardless of equimolar cAMP amounts generated, point to a more complex interaction between β2R and T-cell function [46]. Use of additional β2R signaling pathways most likely accounts for the differing effects of catecholamines during the inflammatory process in RA. These include the modulation of transcription factor NF (nuclear factor)-κB, PKA-independent signal transduction pathways like c-Jun N-terminal kinase/Src family tyrosine kinase Lck, MAPK (mitogen activated protein kinase), and potassium ion channels [47-51]. Additionally, mechanisms that control β2R reactivity apart from GRKs (G protein-coupled receptor kinases) contribute to differences regarding functional consequences upon activation of β2R [52-54].

## Conclusion

In summary, we demonstrate a failure of catecholamines to shift T-cell cytokine responses toward the expected Th2 profile in patients with RA; this failure thus generates a cytokine environment that perpetuates inflammation. The modified catecholaminergic control on cytokine production is specific for the cytokine and cell type studied, resulting in a preserved Th1 profile despite an unimpaired cAMP generation in T cells. Our results further demonstrate that the changes of functional catecholaminergic effects on cytokine production in RA originate from various mechanisms instead of from a single cause.

## Abbreviations

ANOVA = analysis of variance; APC = antigen-presenting cell; β2R = β2-adrenergic receptor; CCP = cyclic citrullinated peptide; CRP = C-reactive protein; DMARD = disease-modifying anti-rheumatic drug; ELISA = enzyme-linked immunosorbent assay; EPI = epinephrine; FCS = fetal calf serum; FITC = fluorescein isothiocyanate; HD = healthy donor; IFN = interferon; mAb = monoclonal antibody; MS = multiple sclerosis; NE = norepinephrine; PBMC = peripheral blood mononuclear cell; PBS = phosphate-buffered saline; PE = phycoerythrin; PGE2 = prostaglandin E2; PI = propidium iodide; PKA = protein kinase A; RA = rheumatoid arthritis; SLE = systemic lupus erythematosus; SNS = sympathetic nervous system; Th1/2 = T helper lymphocyte type 1/2; TNF = tumour necrosis factor.

## Competing interests

The authors declare that they have no competing interests.

## Authors' contributions

MW was responsible for study design and patient recruitment, carried out intracellular cytokine staining and cAMP measurements, and drafted the manuscript. GH carried out the separation of lymphocytes, cell stimulation, and cytokine ELISAs. SB participated in cAMP measurements. RHS participated in statistical analysis, supported figure preparation, and helped with manuscript preparation. UW participated in patient recruitment, statistical analysis, and manuscript preparation. HH participated in the coordination of the study and helped with patient recruitment and manuscript preparation. AK participated in the design of the study and in planning the manuscript. CGOB participated in the design and coordination of the study and helped with the draft of the manuscript. All authors read and approved the final manuscript.

## Figures and Tables

**Table 1:** Clinical characteristics of the healthy control subjects and patients with RA studied

	Patients with RA (*n *= 16)	Control group (*n *= 16)
Gender (female/male)	9/7	7/9
Age, years (range)	63 ± 4.0 (25 to 92)	53 ± 4.2 (26 to 87)
Disease duration, years (range)	11.4 ± 4.8 (1 to 57)	n.a.
Rheumatoid factor-positive	11	n.a.
C-reactive protein (mg/ml)	35.6 ± 9	n.m.
CD4/CD8 ratio	4.4 ± 0.7	3.9 ± 0.5
DAS28-3	4.73 ± 0.44	n.a.

Data are given as means ± standard errors of the mean. DAS28-3, Disease Activity Score using 28 joints and three variables; n.a.; not applicable; n.m.; not measured; RA, rheumatoid arthritis.
